# Quantitative evaluation of blood-tumor-barrier response following focused ultrasound and microbubble treatment in rat glioma: can we improve drug delivery to brain tumors?

**DOI:** 10.1186/2050-5736-3-S1-P8

**Published:** 2015-06-30

**Authors:** Hassaan Ahmed

**Affiliations:** 1 Western University, London, Ontario, Canada

## Background/introduction

The increased interstitial fluid pressure (IFP) from vasogenic edema, which results from a leaky blood-tumor-barrier (BTB), creates a barrier for transvascular drug delivery, particularly larger molecules that are unable to diffuse across and must resort to bulk fluid flow. We have previously used dynamic contrast enhanced-computed tomography (DCE-CT) to quantitatively evaluate the blood-brain-barrier (BBB) response in normal brain - and have shown that focused ultrasound (FUS) and microbubble (MB) treatment in a rat brain demonstrates a 3-4 times transient increase at our optimized parameters. The purpose of this study was to quantitatively evaluate the BTB response following FUS and MB treatment in rat glioma, in the context of improving drug delivery to brain tumors.

## Methods

At 10-15 days before treatment, 1 x 106 C6 glioma cells are surgically implanted using a stereotactic frame into a region in the right cerebral hemisphere of a Wistar rat. The rats were broken into 2 groups before treatment: 1) evaluating the acute response out to 4 h post for tumor and normal hemispheres (n=3), and 2) recovery at 2 h post followed by scans at 24 and 72 h post (n=5). The tumor is transcranially sonicated at previously optimized parameters that demonstrate transient opening in the normal BBB without any vascular or tissue damage - 10 ms burst length with 1 Hz repetition frequency for 120s, with an applied power of 0.5 W, using a 0.563-MHz FUS system. 2 ul/kg of Definity microbubbles are injected simultaneously with the start of the sonication. Axial CT scans as well as the DCE-CT perfusion maps were used to target the tumor. DCE-CT (80 kVp, 250 mA, axial slice thickness = 1.25 mm) was performed using a two phase protocol: 1st phase consisting of continuous 0.5 s rotations for 30 s, and a 2nd phase of 0.5 s rotations at 14.5 s intervals for 150s. 2.5 ml/kg of iodinated contrast agent was injected over 5 seconds simultaneous to the start of each DCE-CT scan. Proprietary CT Perfusion software was used to compute permeability surface area product (PS), cerebral blood flow (CBF), cerebral blood volume (CBV), and mean transit time (MTT) maps using a standard small molecule contrast agent (Isovue ~ 760 Da) that is able to diffuse across the BTB. The BTB PS was also evaluated using a much larger contrast agent (eXia ~ 65,000 Da) that mimics the delivery of agents such as monoclonal antibodies.

## Results and conclusions

Instead of a transient increase in BBB permeability that is seen in the normal brain, FUS and MB demonstrated a gradual decrease in BTB permeability in the hours following treatment (p < 0.05). An acute vascular shutdown, drop in CBF and CBV (p < 0.05), was also observed immediately following treatment, as previously reported in other tumor models. The drop in PS persisted at 24 h post (p < 0.05), returning at 72 h post, whereas the CBF and CBV returned back to baseline levels by 24 h post. A trend of increasing eXia penetration suggests that the decrease in BTB PS alleviates the elevated IFP that results from vasogenic edema and improves the penetration of larger drugs or molecules that are unable to diffuse across the BTB. When plotted against the MTT, which is the inverse of perfusion pressure, BTB PS (isovue) shows a moderate positive correlation (r2 = 0.71), whereas BTB PS (eXia) shows a weak negative correlation (r2 = - 0.50). Our findings indicate that although FUS and MB treatment at our parameters may not necessarily be useful for increasing the delivery of small molecule drugs, it may be more useful for increasing the delivery of larger molecules where the IFP is a barrier.

**Figure 1:**
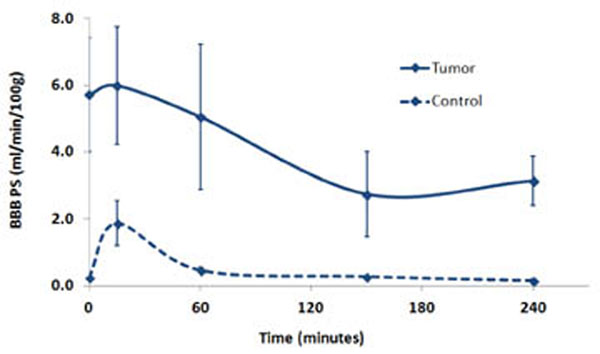
Acute blood-tumor-barrier (BTB) and blood-brain-barrier (BBB) permeability surface-area product (PS) time curves following focused ultrasound (FUS) and microbubble (MB) treatment for the tumor and contralateral hemispheres (n=3)

**Figure 2:**
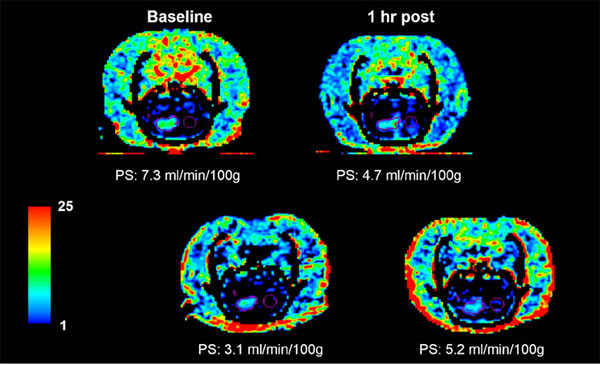
Blood-tumor-barrier (BTB) permeability surface-area product (PS) maps at baseline,1, 24, and 72 h following focused ultrasound (FUS) and microbubble (MB) treatment

**Figure 3:**
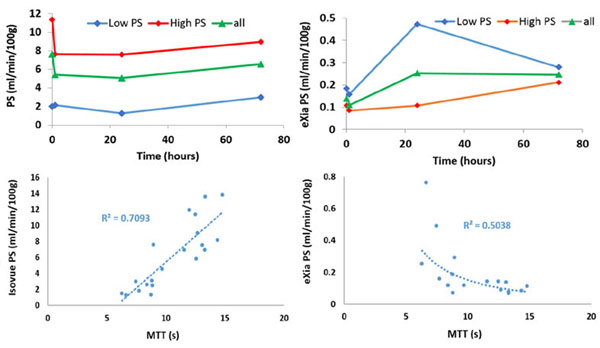
Comparison of isovue (~760 Da), and eXia (~65,000 Da) blood-tumor-barrier (BTB) permeability surface-area product (PS) plotted against the mean-transit-time (MTT) as measured with the standard iodine diffusable iodine contrast (Isovue)

